# New Platinum(IV) and Palladium(II) Transition Metal Complexes of s-Triazine Derivative: Synthesis, Spectral, and Anticancer Agents Studies

**DOI:** 10.1155/2019/9835745

**Published:** 2019-02-17

**Authors:** Fatima A. I. Al-Khodir, Hana M. A. Abumelha, Tarfah Al-Warhi, S. A. Al-Issa

**Affiliations:** Department of Chemistry, College of Science, Princess Nourah Bint Abdulrahman University, Saudi Arabia

## Abstract

New Pd(II) and Pt(IV) triazine complexes [Pt_3_(**L**^**1**^)_2_(Cl)_9_(H_2_O)_3_].3Cl.3H_2_O (**1**), [Pt_3_(**L**^**2**^)_2_(Cl)_9_(H_2_O)_3_].3Cl (**2**), [Pt_3_(**L**^**3**^)_2_(Cl)_9_(H_2_O)_3_].3Cl (**3**), [Pt_2_(**L**^**4**^)_2_(Cl)_6_(H_2_O)_2_] .2Cl.4H_2_O (**4**), [Pd_3_(**L**^**1**^)_2_(H_2_O)_6_] .3Cl_2_ (**5**), [Pd_3_(**L**^**2**^)_2_(H_2_O)_6_].3Cl_2_ (**6**), [Pd_3_(**L**^**3**^)_2_(H_2_O)_6_].3Cl_2_ (**7**), and [Pd_2_(**L**^**4**^)_2_(H_2_O)_4_].2Cl_2_ (**8**) were synthesized and well characterized using elemental analyses, molar conductance, IR, UV-Vis, magnetic susceptibility, ^1^H, ^13^C-NMR spectra, and thermal analyses. These analyses deduced that the** L**^**1**^,** L**^**2**^, and** L**^**3**^ ligands act as tridentate forming octahedral geometry with Pt(IV) metal ions and square planar geometry in case of Pd(II) complexes but the** L**^**4**^ ligand acts as bidentate chelate. The molar conductance values refer to the fact that all the prepared s-triazine complexes have electrolyte properties which are investigated in DMSO solvent. Surface morphology behaviors of prepared complexes have been scanned using TEM. The crystalline behavior of triazine complexes has been checked based on X-ray powder diffraction patterns. The antimicrobial activity of the free ligands and their platinum(IV) and palladium(II) complexes against the species* Staphylococcus aureus* (G+),* Escherichia coli* (G−),* Aspergillus flavus*, and* Candida albicans *has been carried out and compared with the standard one. The coordination of ligands towards metal ions makes them stronger bacteriostatic agents, thus inhibiting the growth of bacteria and fungi more than the free ligands. The cytotoxic assessment IC_50_ of the free ligands and its platinum(IV) complexes* in vitro* against human colon and lung cancer cell lines introduced a promising efficiency.

## 1. Introduction

The s-triazine and its derivatives have a wide range of pharmaceutical benefits: antiviral, antimalarial, antibacterial, anti-inflammatory, antileukemia, anticancer, and anti-HIV activities [1–4]. Many triazine analogues are utilized as a building block for the construction of multisite ligand systems [5, 6]. A number of triazine analogues were tested for ion extraction of metal ions which have been reviewed [7, 8]. Some triazine derivatives with pyrazole, functioning at the least conventional habiliments, are screened and identified as potential inhibitors of photosynthetic electron transport [9]. In literature survey, it was refereed that the organic s-triazine's derivative compounds and transition metal complexes have been found to be effective in the field of nonlinear optical activity (NLO) [10–12] that can act as an auxiliary acceptor in NLO chromophores. Further advantages in considering the s-triazine as central moiety are its symmetric nature by which it will be possible to chemically tune its NLO nature by mono- or disubstitution [13, 14]. 1,3,5-Triazine is considered to be a remarkable in supramolecular chemistry since it can take part in all types of interactions, namely, coordination, hydrogen bonds, electrostatic and charge-transfer attractions, and aromatic-stacking interactions [15]. Triazine derivatives have been widely used in several fields such as herbicide [16, 17]. In materials chemistry 1,3,5-triazine derivatives have been used as acceptors in star-shaped systems [18], liquid crystals [19], redox active chromophores [20], photovoltaic devices [21], and blue phosphorescent [22]. Also, it was used as functional materials [23], catalysts [24], absorption of surfactants [25], nanoporous membranes for desalination [26], and cathodes for lithium batteries [27, 28].

**Figure 1:**
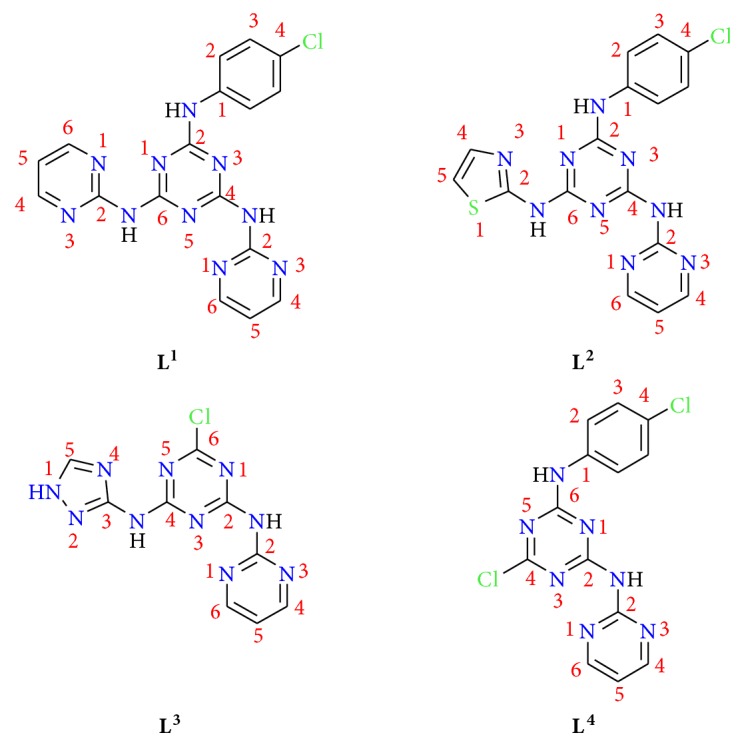
Structures of prepared triazine ligands [29].*** L***^***1***^*= N*^*2*^*-(4-chlorophenyl)-N*^*4*^*,N*^*6*^*-di(pyrimidin-2-yl)-1,3,5-triazine-2,4,6-triamine*.*** L***^***2***^*= N*^*2*^*-(4-chlorophenyl)-N*^*4*^*,N*^*6*^*-di(pyrimidin-2-yl)-1,3,5-triazine-2,4,6-triamine*.*** L***^***3***^*=6-chloro-N*^*2*^*-(pyrimidin-2-yl)-N*^*4*^*-(1H-1,2,4-triazol-3-yl)-1,3,5-triazine-2,4-diamine*.*** L***^***4***^*= 6-chloro-N*^*2*^*-(4-chlorophenyl)-N*^*4*^*-(pyrimidin-2-yl)-1,3,5-triazine-2,4-diamine)*.

In connection of our previously work [29], this article aimed to synthesized new platinum(IV) and palladium(II) complexes with four triazine ligands (Figure 1) and checked the biological property and anticancer significance.

## 2. Materials and Methods

### 2.1. Chemicals

The PtCl_4_ and PdCl_2_ salts were received from Sigma-Aldrich Chemical Corporation, St. Louis, Mo, USA.

### 2.2. Synthesis of Trisubstituted s-Triazine Derivative Ligands

**Figure 2:**
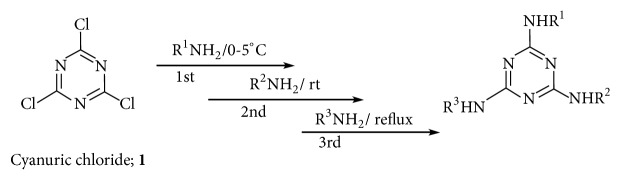
Synthesis of trisubstituted triazine derivatives.

Synthesis of* N*^*2*^*,N*^*4*^*,N*^*6*^-triaryl-1,3,5-triazine-2,4,6-triamine primary nucleus (Figure 2) was carried out as described in literature [30, 31]. The synthesis of** L**^**1**^,** L**^**2**^,** L**^**3**^, and** L**^**4**^ triazine ligands was carried out according to our previously work [29],** L**^**1**^ prepared by stirring the mixtures of 2-aminopyrimidine with 6-chloro-N^2^-(4-chlorophenyl)-N^4^-di(pyrimidin-2-yl)-1,3,5-triazine-2,4-diamine in dioxane at refluxed temperature;** L**^**2**^ prepared by stirring the mixtures of 2-aminothiazole with 6-chloro-N^2^-(4-chlorophenyl)-N^4^-(pyrimidin-2-yl)-1,3,5-triazine-2,4-diamine in dioxane at refluxed temperature;** L**^**3**^ prepared by stirring the mixtures of 2-aminopyrimidine with 4,6-dichloro-N^2^-(1H-1,2,4-triazol-3-yl)-1,3,5-triazin-2-amine in dioxane 50°C;** L**^**4**^ prepared by stirring the mixtures of 2-aminopyrimidine with 4,6-dichloro-N-( 4-chlorophenyl)-1,3,5-triazin-2-amine in dioxane 50°C.

### 2.3. Synthesis of Pt(IV) and Pd(II) Complexes

A hot methanolic solution of the metal chloride (Pt(IV) and Pd(II)) (1 mM) was added to the hot methanolic solution of ligands (**L**^**1**^,** L**^**2**^,** L**^**3**^, or** L**^**4**^) (1 mM). The mixed solutions were stirred and refluxed at 70°C for 6 hrs. The colored precipitates thus separated out were washed with methanol and dried in* vacuo*.

### 2.4. Instrumentals

**Table:** 

No.	Type of analysis	Model of the instruments
(i)	Elemental analyses	Perkin Elmer CHN 2400 (USA)
(ii)	Metal ions	gravimetrically
(iii)	Melting point	MPS10–120
(iv)	Molar conductivities	Jenway 4010 conductivity meter
(v)	Infrared spectra	Bruker Alpha FTIR Spectrophotometer
(vi)	UV-Vis absorption spectra	UV2 Unicam UV/Vis Spectrophotometer
(vii)	Magnetic moments	Magnetic Susceptibility Balance, Sherwood Scientific, Cambridge Science Park, Cambridge, England
(viii)	^1^H,^13^C-NMR spectra	Oxford YH-300 NMR spectrometer
(ix)	Mass spectra	70 eV using AEI MS 30 mass spectrometer
(x)	Thermal studies TG/DTG	Mettler Toledo AG thermogravimetric analyzer
(xi)	SEM	Quanta FEG 250 equipment
(xii)	XRD	X 'Pert PRO PANanalytical X-ray powder diffraction
(xiii)	TEM	JEOL 100s microscopy

### 2.5. Antimicrobial Study

Antimicrobial evaluations of the investigated samples were assessed by a modified Kirby-Bauer disc diffusion method [32, 33].

### 2.6. Anticancer Study

All tested samples were checked against human colon and lung cancer cell line by using neutral red (NR) technique [34].

## 3. Results and Discussion

### 3.1. Microanalytical and Physical Data

**Figure 3:**
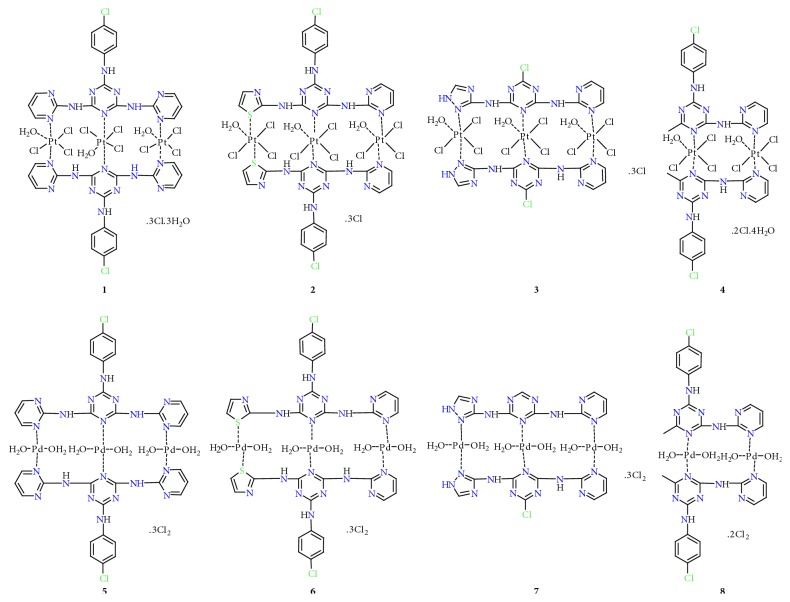
Suggested structures of Pt(IV) and Pd(II) complexes.

All the platinum(IV) and palladium(II) s-triazine derivative complexes were obtained as colored solids by the reaction of ligands (**L**^**1**^,** L**^**2**^,** L**^**3**^, and** L**^**4**^) with anhydrous metal chloride salts (PtCl_4_ and PdCl_2_). The experimental of elemental analyses of the ligands and their metal complexes (Table 1) are in good agreement with the calculated data. The ligands and their metal (IV/II) complexes are stable at room temperature and soluble in common organic solvents such as (DMSO and DMF). According to the elemental analysis and spectroscopic assignments, the chelating sites and geometry have been suggested and are displayed in Figure 3. The molar conductance of both free s-triazine derivative ligands and their Pt(IV) and Pd(II) complexes in 10^−3^ M of DMSO solution is in the range of 64.7–139.3 *μ*S, which reveals the electrolytic behavior of the complexes [35]. Melting points of all complexes have values more than >300°C due to thermal stability properties.

### 3.2. FT-IR Spectra

**Figure 4:**
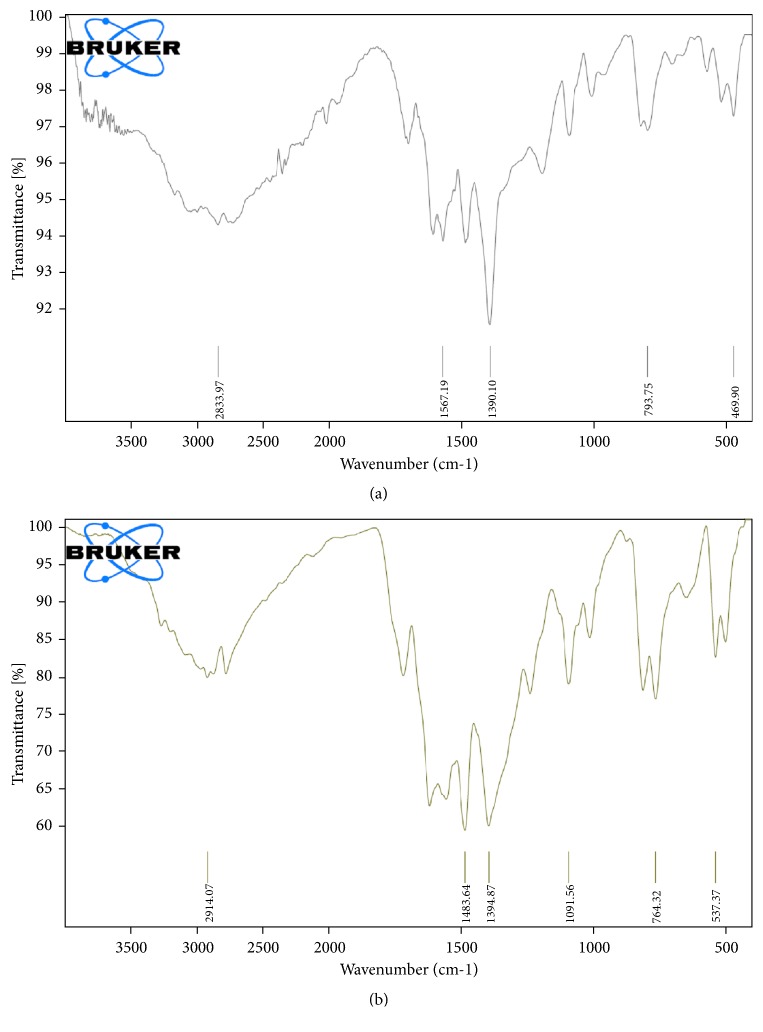
FT-IR spectra of (a) [Pt_2_(**L**^**4**^)_2_(Cl)_6_(H_2_O)_2_].2Cl.4H_2_O (**4**) and (b) [Pd_3_(**L**^**2**^)_2_ (H_2_O)_6_].3Cl_2_ (**6**).

Peaks at 1620, 1560, 1485, 740, and 627 cm^−1^ present in** L**^**1**^,** L**^**2**^,** L**^**3**^, and** L**^**4**^ s-triazine derivatives ligands can be assigned for the C=N_pyrimidine_, C=C, C=N, C–S, and C–Cl stretching vibrations. The FT-IR spectra of the ligands show a strong-to-medium strong bands at 1488 cm^−1^ (**L**^**1**^), 1485 cm^−1^ (**L**^**2**^), 1510 cm^−1^ (**L**^**3**^), and 1484 cm^−1^ (**L**^**4**^) which are assigned to *ν*(C=N) group of triazine [36]. Infrared spectral data of the** 1**–**8** complexes (Table 2; Figure 4) usually a lot of valuable information is provided about the coordination mechanism. The free ligands which exhibit a band at 1623 cm^−1^ (**L**^**1**^), 1619 cm^−1^ (**L**^**2**^), 1621 cm^−1^ (**L**^**3**^), and 1619 cm^−1^ (**L**^**4**^) are assigned to *ν*(C=N) of pyrimidine and triazole rings. In case of complexes, this band is shifted to 1698–1667 cm^−1^ region attributed to nitrogen atom of (C=N) coordination to metal ion. The ligands shows a medium strong band at 1510–1484 cm^−1^, which is characteristic of the *ν*(C=N) group in s-triazine [37, 38]. This band shifted to lower frequency of 1396–1382 cm^−1^ upon complexation which indicates that triazine ring nitrogen is one of the coordinating atoms in the ligand [38]. The *ν*(N–H) stretching frequency of pyrimidine/triazole rings exhibited at 3260–3112 cm^−1^ was shifted to lower wavenumbers after complexation due to the reduction of lone pair repulsive forces on the nitrogen atoms [39]. In the FT-IR spectra of complexes, the medium-weak bands appeared at 570–440 cm^−1^ regions which can be assigned to *ν*(M–N) [40] and confirm the interaction between metal and ligand.

### 3.3. Electronic and Magnetic Studies

The electronic spectra of [Pt_3_(**L**^**1**^)_2_(Cl)_9_(H_2_O)_3_].3Cl.3H_2_O (**1**), [Pt_3_(**L**^**2**^)_2_(Cl)_9_(H_2_O)_3_].3Cl (**2**), [Pt_3_(**L**^**3**^)_2_(Cl)_9_(H_2_O)_3_].3Cl (**3**), and [Pt_2_(**L**^**4**^)_2_(Cl)_6_(H_2_O)_2_] .2Cl.4H_2_O (**4**) complexes which displayed charge-transfer transitions may interfere and prevent the observation of all the expected bands [41, 42].The distinct bands at 300–311 and 337–396 cm^−1^ are attributed to a combination of metal ligand charge transfer (M→L_CT_) and d–d transition band. The other weak band at 429–437 cm^−1^ is attributed to combination of N→Pt(IV) metal charge transfer (L*π*→M_CT_) and d–d transition bands. The Pt(IV) complexes are found to be diamagnetic character, so the Pt(IV) complexes must be octahedral geometry. The Pt(IV) is d^6^ system and four bands are expected due to ^1^A_1g_→  ^3^T_1g_, ^1^A_1g_→  ^3^T_2g_, ^1^A_1g_→  ^1^T_1g_, and ^1^A_1g_→  ^1^T_2g_ transitions. The shift to lower frequency after complexation is due to the binding between Pt(IV) ion nitrogen atom of triazine, pyrimidine, thiazole, and triazole rings. Palladium(II) complexes have diamagnetic properties. The electronic absorption spectra of palladium(II) complexes have distinguished bands at (300 nm), (312 and 352 nm), (309, 334 and 391 nm), and (296, and 339 nm) for** L**^**1**^,** L**^**2**^,** L**^**3**^, and** L**^**4**^ ligands, respectively, due to Pd-L^n^_CT_ charge-transfer transitions.

### 3.4. ^1^H, ^13^C-NMR Spectra

*Complex* ***1***. ^1^H-NMR (DMSO-*d*_6_): *δ* = 7.26 (t, H,* J =* 4.5 Hz, pyrimidine* C*_*5*_*H*), 7.30 (t, H,* J =* 4.5 Hz, pyrimidine* C*_*5*_*H*), 7.34 (d, 2H,* J =* 4.5 Hz,* p*-Chloroaniline* C*_*3*_*H*), 7.39 (d, 2H,* J =* 9.9 Hz,* p*-Chloroaniline* C*_*2*_*H*), 8.81 (d, 2H,* J =* 4.5 Hz, pyrimidine* C*_*4,6*_*H*), 8.91 (d, 2H,* J =* 4.5 Hz, pyrimidine* C*_*4,6*_*H*), 9.82 (s, 1H, NH), 11.19 (s, 1H, NH), 12.06 (s, 1H, NH). ^13^C-NMR (DMSO-*d*_6_): *δ* = 111.4, 113.8, 117.9, 123.9, 130.7, 140.1, 144.1, 147.5, 153.1, 157.1, 159.2, 163.4, 163.9, 166.2, and 169.7 (Ar-C, C=C, C=N).

*Complex* ***2***. ^1^H-NMR (DMSO-*d*_6_): *δ* = 7.34 (d, 2H,* J =* 7.2 Hz, thiazole* C*_*5*_*H*), 7.38 (t, 4H,* J =* 4.5 Hz, pyrimidine* C*_*5*_*H*), 7.44 (d, 4H,* J =* 4.5 Hz,* p*-Chloroaniline* C*_*3*_*H*), 7.77 (d, 4H,* J =* 9.9 Hz,* p*-Chloroaniline* C*_*2*_*H*), 7.86 (d, 2H,* J =* 6.3 Hz, thiazole* C*_*4*_*H*), 8.09 (d, 4H,* J =* 4.5 Hz, pyrimidine* C*_*4,6*_*H*), 9.51 (s, 2H, NH), 11.18 (s, 2H, NH), 12.08 (s, 2H, NH). ^13^C-NMR (DMSO-*d*_6_): *δ* = 112.5, 114.0, 114.9, 123.2, 132.5, 145.2, 151.4, 152.9, 155.4, 157.0, 158.8, 162.0, 163.5, and 172.2 (Ar-C, C=C, C=N, C-S).

*Complex* ***3***. ^1^H-NMR (DMSO-*d*_6_): *δ* = 7.23 (t, 2H,* J =* 4.5 Hz, pyrimidine* C*_*5*_*H*), 7.32 (s, 2H, triazole* C*_*5*_*H*), 8.45 (d, 4H,* J =* 4.5 Hz, pyrimidine* C*_*4,6*_*H*), 8.91 (s, 2H, NH), 11.22 (s, 2H, NH), 11.46 (s, 1H, NH). ^13^C-NMR (DMSO-*d*_6_): *δ* = 115.8, 142.8, 146.5, 155.0, 156.2, 159.2, 163.8, 163.9, and 167.1 (Ar-C, C=C, C=N, C-Cl).

*Complex* ***4***. ^1^H-NMR (DMSO-*d*_6_): *δ* = 7.35 (t, 4H,* J =* 4.5 Hz, pyrimidine* C*_*5*_*H*), 7.39 (d, 4H,* J =* 4.5 Hz,* p*-Chloroaniline* C*_*3*_*H*), 7.44 (d, 4H,* J =* 9.9 Hz,* p*-Chloroaniline* C*_*2*_*H*), 7.76 (d, 4H,* J =* 4.5 Hz, pyrimidine* C*_*4,6*_*H*), 9.50 (s, 2H, NH), 11.19 (s, 2H, NH). ^13^C-NMR (DMSO-*d*_6_): *δ* = 111.9, 118.0, 123.1, 132.4, 144.4, 147.9, 153.5, 156.8, 161.8, 166.6, and 172.1 (Ar-C, C=C, C=N, C-Cl).

*Complex* ***5***. ^1^H-NMR (DMSO-*d*_6_): *δ* = 7.32 (t, 2H,* J =* 4.5 Hz, pyrimidine* C*_*5*_*H*), 7.35 (t, 2H,* J =* 4.5 z, pyrimidine* C*_*5*_*H*), 7.41 (d, 4H,* J =* 4.5 Hz,* p*-Chloroaniline* C*_*3*_*H*), 7.46 (d, 4H,* J =* 9.9 Hz,* p*-Chloroaniline* C*_*2*_*H*), 8.57 (d, 4H,* J =* 4.5 Hz, pyrimidine* C*_*4,6*_*H*), 7.79 (d, 4H,* J =* 4.5 Hz, pyrimidine* C*_*4,6*_*H*), 9.55 (s, 2H, NH), 11.19 (s, 2H, NH), 11.20 (s, 2H, NH). ^13^C-NMR (DMSO-*d*_6_): *δ* = 113.1, 115.1, 120.1, 123.8, 129.4, 141.7, 147.0, 149.8, 152.4, 157.7, 159.6, 163.1, 163.9, 165.8, and 171.1 (Ar-C, C=C, C=N).

*Complex* ***6***. ^1^H-NMR (DMSO-*d*_6_): *δ* = 7.33 (d, 2H,* J =* 7.2 Hz, thiazole* C*_*5*_*H*), 7.36 (t, 4H,* J =* 4.5 Hz, pyrimidine* C*_*5*_*H*), 7.45 (d, 4H,* J =* 4.5 Hz,* p*-Chloroaniline* C*_*3*_*H*), 7.78 (d, 4H,* J =* 9.9 Hz,* p*-Chloroaniline* C*_*2*_*H*), 7.81 (d, 2H,* J =* 6.3 Hz, thiazole* C*_*4*_*H*), 8.12 (d, 4H,* J =* 4.5 Hz, pyrimidine* C*_*4,6*_*H*), 9.55 (s, 2H, NH), 11.20 (s, 2H, NH), 12.02 (s, 2H, NH). ^13^C-NMR (DMSO-*d*_6_): *δ* = 110.2, 113.1, 115.8, 123.8, 131.8, 146.1, 150.3, 152.1, 155.0, 156.6, 158.1, 161.2, 163.1, and 173.4 (Ar-C, C=C, C=N, C-S).

*Complex* ***7***. ^1^H-NMR (DMSO-*d*_6_): *δ* = 7.21 (t, 2H,* J =* 4.5 Hz, pyrimidine* C*_*5*_*H*), 7.39 (s, 2H, triazole* C*_*5*_*H*), 8.46 (d, 4H,* J =* 4.5 Hz, pyrimidine* C*_*4,6*_*H*), 8.92 (s, 2H, NH), 11.20 (s, 2H, NH), 11.48 (s, 1H, NH). ^13^C-NMR (DMSO-*d*_6_): *δ* = 116.5, 141.9, 147.4, 154.4, 155.9, 159.8, 163.1, 164.5, and 168.8 (Ar-C, C=C, C=N, C-Cl).

*Complex* ***8***. ^1^H-NMR (DMSO-*d*_6_): *δ* = 7.33 (t, 4H,* J =* 4.5 Hz, pyrimidine* C*_*5*_*H*), 7.36 (d, 4H,* J =* 4.5 Hz,* p*-Chloroaniline* C*_*3*_*H*), 7.46 (d, 4H,* J =* 9.9 Hz,* p*-Chloroaniline* C*_*2*_*H*), 7.81 (d, 4H,* J =* 4.5 Hz, pyrimidine* C*_*4,6*_*H*), 9.55 (s, 2H, NH), 11.20 (s, 2H, NH). ^13^C-NMR (DMSO-*d*_6_): *δ* = 113.5, 119.2, 122.9, 130.5, 145.5, 149.4, 153.1, 157.4, 162.7, 165.6, and 171.8 (Ar-C, C=C, C=N, C-Cl).

The ^1^H-NMR spectral data of the synthesized Pt(IV) and Pd(II) complexes have been shifted to downfield because of formation metal chelating through the nitrogen atoms of triazine, pyrimidine, thiazole, and triazole rings.

### 3.5. Thermogravimetric Studies

**Figure 5:**
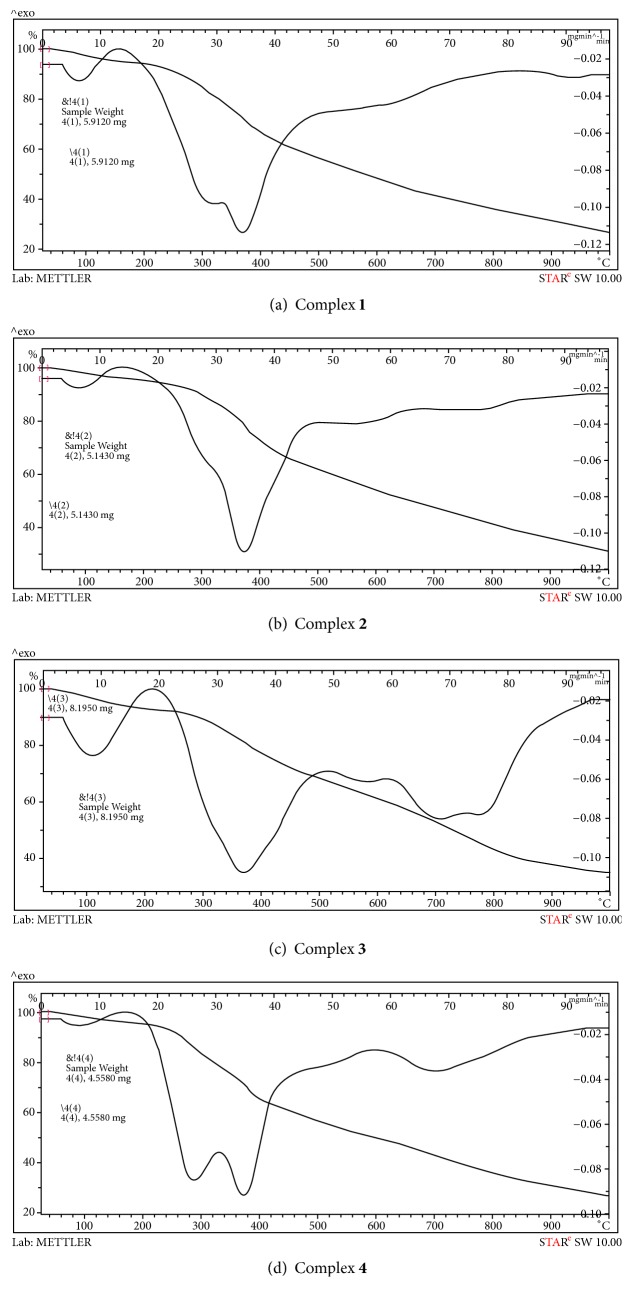
TGA-DTG curves of Pt(IV) complexes** 1**–**4**.

**Figure 6:**
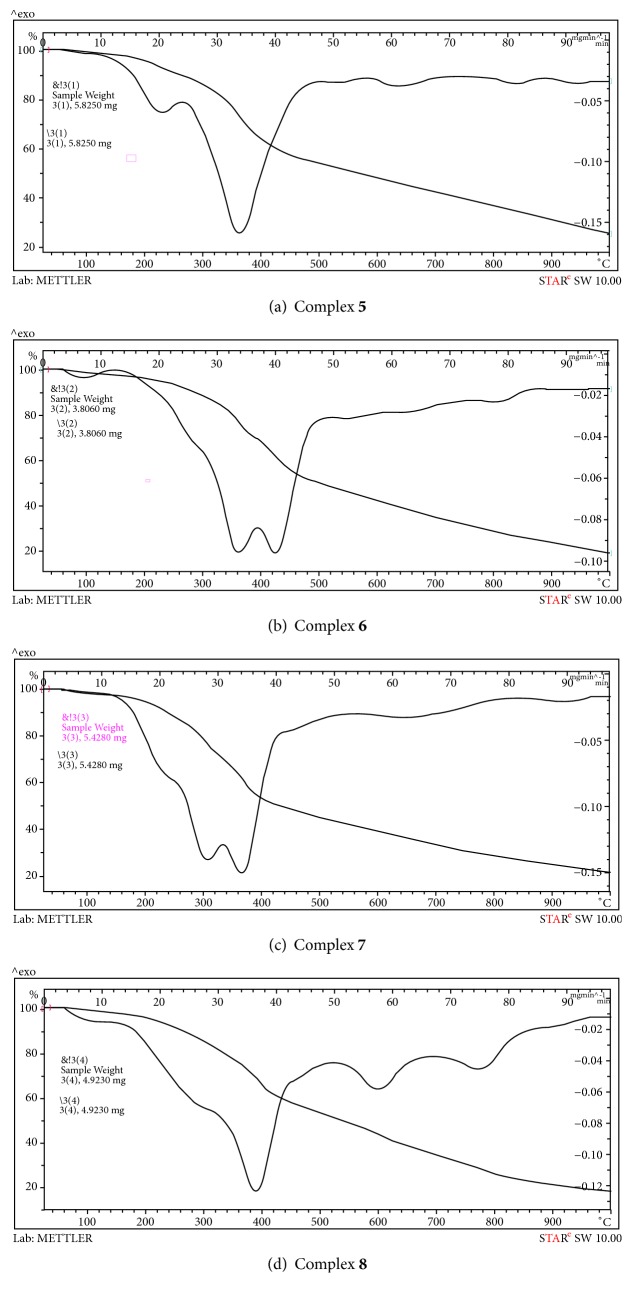
TGA-DTG curves of Pd(II) complexes** 5**–**8**.

Thermal analyses (TG-DTG) were performed under N_2_ atmosphere. The thermogravimetric and differential thermogravimetric curves of the synthesized Pt(IV) (**1**–**4**) and Pd(II) (**5**–**8**) complexes are shown in Figures 5 and 6.  Table 3 refereed to the thermal decomposition assignments of all complexes from room temperature till 1000°C.

### 3.6. X-Ray Diffraction Spectra

**Figure 7:**
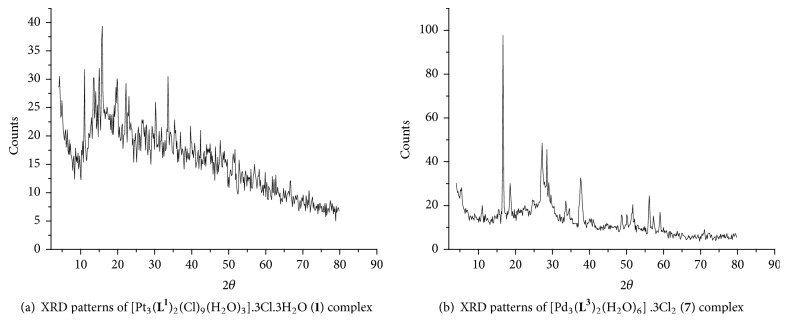


XRD diffraction patterns of the solid Pt(IV) and Pd(III) triazine complexes have been displayed in Figure 7. The diffraction patterns of new Pt(IV) and Pd(II) complexes at 2*θ* values are (11.094, 13.585, 15.041, 15.840, 19.929, 22.276, 23.198, 30.360, 33.693, 39.780, 47.720°), (5.082, 12.894, 19.845, 29.786°), (12.861, 16.426, 17.438, 19.695, 22.031, 26.390, 35.265°), (13.078, 19.773, 29.893, 35.342, 45.307°), (16.712, 27.173, 28.562, 31756, 37.958, 45.472, 56.157, 57.440, 59.071°), (4.948, 10.051, 13.820, 16.705, 17.357, 19.608, 20.512, 24.738, 26.567, 27.247, 29.786, 37.806, 56.168, 57.332, 59.199°), (5.527, 11.110, 16.704, 18.605, 27.263, 28.547, 29.090, 33.708, 37.678, 51.690, 56.690, 56.193, 57.367, 59.096, 79.472°), and (16.839, 27.384, 28.680, 37.851, 48.826, 50.279, 56.287, 57.573, 59.194°) for the complexes** 1**–**8**, respectively. The particle size was estimated using Scherrer's equation [43]. The XRD patterns due to metallic platinum are agreement with JCPDS PDF card no. 04-0802 standard card [44] with (111), (200), (220) planes, respectively. Powder XRD patterns of Pd(II) complexes are shown in Figure 7. These spectra included distinguish patterns at 2*θ* = 37.678, 51.690, 59.096, and 79.472° assigned to (111), (200), (220), and (311) of Pd metal with fcc structure matching with JCPDS file no. 87-0638 [45]. This result confirms the presence of metallic Pd with fcc structure. The grain sizes of platinum(IV) and palladium(II) complexes are existed within 42–50 and nm according to highest distinguish peaks.

### 3.7. Scanning and Transmission Electron Microscopes

**Figure 8:**
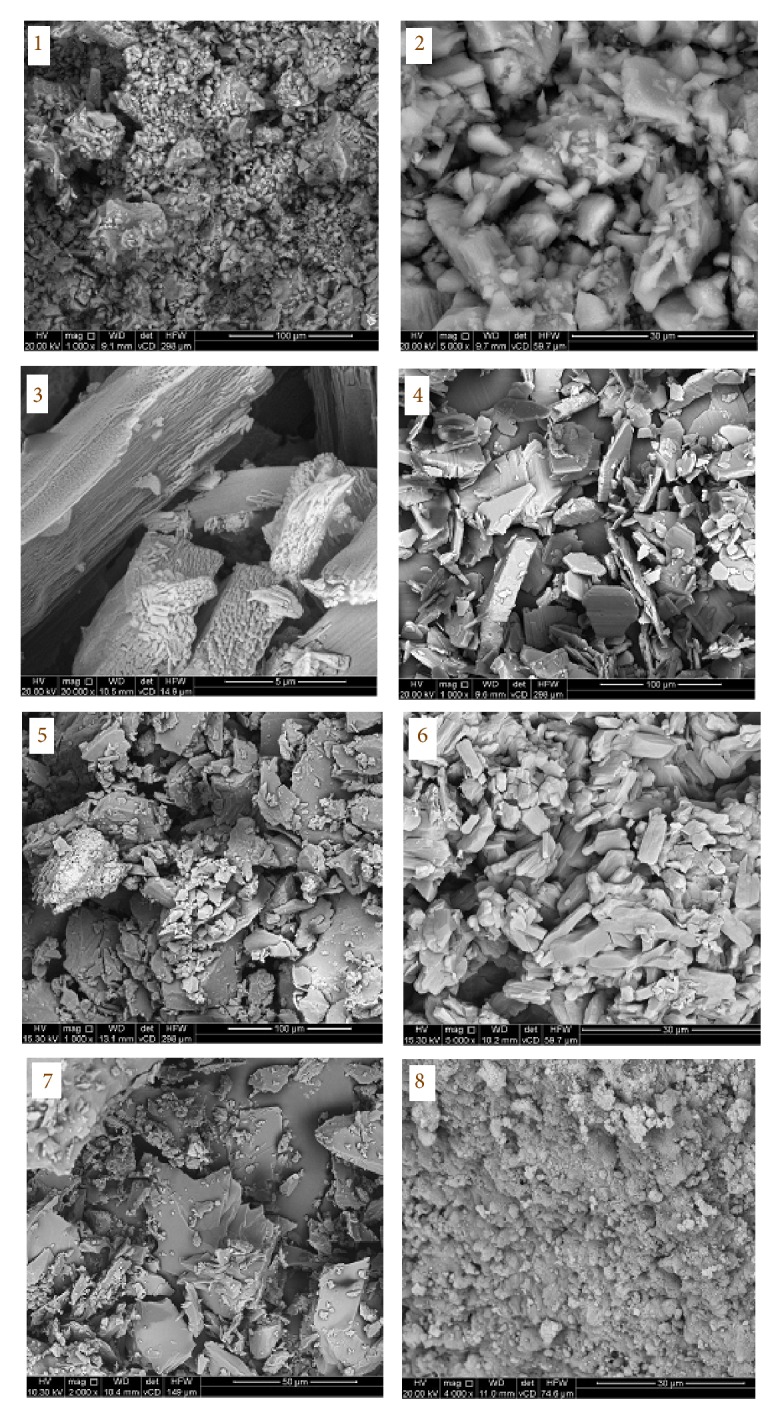
SEM photos of Pt(IV) and Pd(II) complexes** 1**–**8**.

The SEM photos of Pt(IV) and Pd(II) complexes** 1**–**8** are shown in Figure 8. These images reveal that the surface of all complexes is homogeneous with various morphological view because of the role of Pt(IV) and Pd(II) metal ions in the rearrangement of grains.

**Figure 9:**
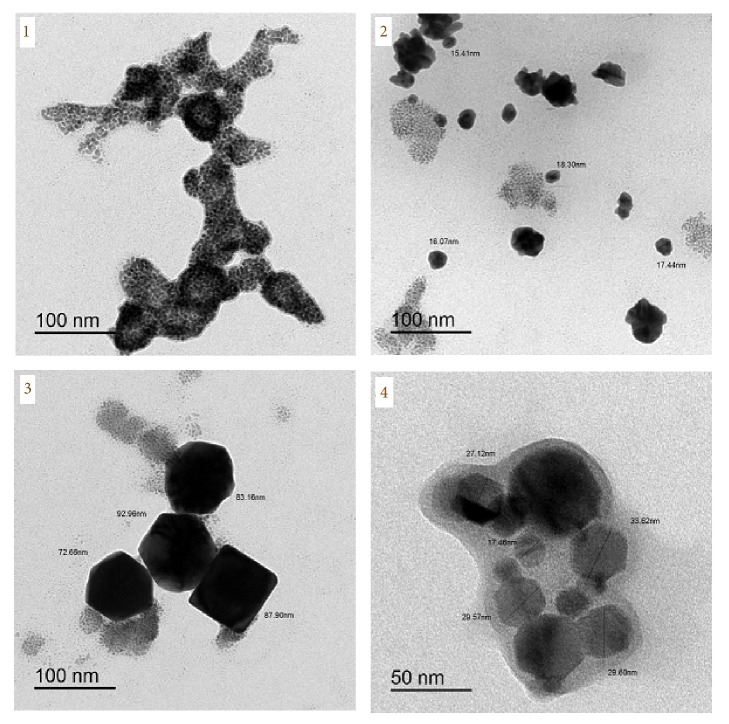
TEM photos of Pt(IV) complexes** 1**–**4**.

According to the TEM technique (Figure 9), the average of particle size of platinum(IV) complexes existed within 15–92 nm.

### 3.8. Biological Studies

#### 3.8.1. Antibacterial Assessments

Table 4 refers to the antibacterial activity of the free triazine ligands (**L**^**1**^,** L**^**2**^,** L**^**3**^, and** L**^**4**^) comparable with its platinum(IV) and palladium(II) complexes (**1**–**8**) against* Staphylococcus aureus* (G+),* Escherichia coli* (G−), and fungi (*Aspergillus flavus* and* Candida albicans*). All complexes beside the four free ligands which have not any significant inhibitory against both respected fungi except for complexes of** 1**,** 3**,** 5**,** 7,** and** 8** have moderate inhibitory against* Aspergillus flavus*. All complexes have a moderate bacterial inhibitory in comparison with ampicillin standard drug. The variation in the activity of different metal complexes against different microorganisms depends on either the impermeability of the cells of the microbes or the differences in ribosomes in microbial cells [46, 47].

#### 3.8.2. Anticancer Assessments

**Figure 10:**
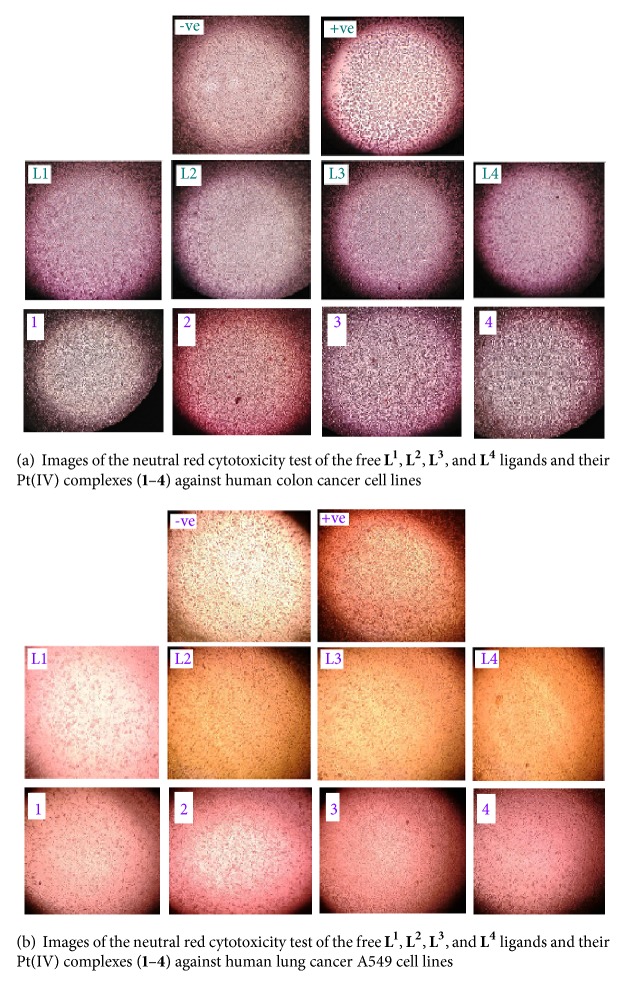


Table 5 and Figure 10 refer to the IC_50_ results of the free triazine ligands and its Pt(IV) complexes. From these data, it is clearly deduced that the [Pt_3_(**L**^**3**^)_2_(Cl)_9_(H_2_O)_3_].3Cl (**3**) complex has an efficiency against human colon and human lung cancer A549 cell lines rather than its corresponding free** L**^**3**^ ligand.

## Figures and Tables

**Table 1:** Microanalytical and physicochemical data of ligands and their complexes.

Compounds*∗*	Color	Λ(*μ*S)	Elemental analyses found(Calc.)				Yield, %
			%C	%H	%N	%M	
**1**	Yellow	126.8	(21.44)	(2.01)	(14.71)	(30.73)	77
			21.32	1.96	14.57	30.66	

**2**	Brown	70.6	(20.66)	(1.63)	(13.55)	(31.46)	72
			20.54	1.54	13.50	31.32	

**3**	Green	64.7	(13.13)	(1.22)	(17.02)	(35.55)	71
			13.09	1.18	17.00	35.50	

**4**	Pale yellow	95.6	(22.64)	(2.19)	(14.22)	(28.29)	69
			22.56	2.13	14.16	28.11	

**5**	Dark brown	139.3	(28.64)	(2.69)	(19.65)	(22.39)	75
			28.56	2.57	19.54	22.31	

**6**	Red brown	82	(26.77)	(2.53)	(17.56)	(22.24)	70
			26.71	2.51	17.49	22.19	

**7**	Brownish green	106.7	(17.70)	(2.15)	(22.94)	(26.14)	74
			17.65	2.09	22.90	26.11	

**8**	Brown	122.3	(30.49)	(2.56)	(19.15)	(20.78)	71
			30.41	2.49	19.12	20.69	

*∗* [Pt_3_(**L**^**1**^)_2_(Cl)_9_(H_2_O)_3_].3Cl.3H_2_O (**1**), [Pt_3_(**L**^**2**^)_2_(Cl)_9_(H_2_O)_3_].3Cl (**2**), [Pt_3_(**L**^**3**^)_2_(Cl)_9_(H_2_O)_3_].3Cl (**3**), [Pt_2_(**L**^**4**^)_2_(Cl)_6_(H_2_O)_2_] .2Cl.4H_2_O (**4**), [Pd_3_(**L**^**1**^)_2_(H_2_O)_6_].3Cl_2_ (**5**), [Pd_3_(**L**^**2**^)_2_(H_2_O)_6_].3Cl_2_ (**6**), [Pd_3_(**L**^**3**^)_2_(H_2_O)_6_] .3Cl_2_ (**7**) and [Pd_2_(**L**^**4**^)_2_(H_2_O)_4_].2Cl_2_ (**8**).

**Table 2:** FT-IR spectral band assignments of **L**^**1**^, **L**^**2**^, **L**^**3**^, and **L**^**4**^ ligands and their complexes.

Compounds	FTIR spectral assignments (cm^−1^)				
	*ν*(N–H)	*ν*(C=N)_aromatic_	*ν*(C=C)_aromatic_	*ν*(C=N)_triazine_	*ν*(M–N)
**L** ^**1**^	3249-3112	1623	1559	1488	-

**L** ^**2**^	3260-3142	1619	1555	1485	-

**L** ^**3**^	3251-3156	1621	1586	1510	-

**L** ^**4**^	3244-3150	1619	1574	1484	-

**1**	3200	1679	1560	1385	545, 447

**2**	-	1667	1537	1383	536, 441

**3**	-	1698	1585	1382	570, 470

**4**	-	1695	1567	1390	530, 469

**5**	-	1689	1530	1394	537, 440

**6**	-	1695	1557	1396	540, 463

**7**	-	1698	1550	1391	537, 467

**8**	-	1691	1537	1393	537, 463

**Table 3:** Thermo gravimetric data of Pt(IV) and Pd(II) triazine complexes.

Complexes	DTG_max_	Total weight loss		Total residual	
		Weight loss, %	Assignments	Residue, %	Assignments
**1**	100	74	3H_2_O uncoord	26	PtO_2_ + Few carbons
	300, 380, 600		2**L**^**1**^+6Cl_2_		

**2**	100	70	3H_2_O coord	30	PtO_2_ + Few carbons
	310, 380, 580		2**L**^**2**^+6Cl_2_		

**3**	100	68	3H_2_O coord	32	PtO_2_ + Few carbons
	370, 580, 700		2**L**^**3**^+6Cl_2_		

**4**	100	72	4H_2_O uncoord	28	PtO_2_ + Few carbons
	280, 380, 700		2**L**^**4**^+4Cl_2_**+**2H_2_O		

**5**	230, 360, 620	75	2**L**^**1**^+3Cl_2_+6H_2_O	25	PdO + Few carbons

**6**	360, 420, 800	75	2**L**^**2**^+3Cl_2_+6H_2_O	25	PdO + Few carbons

**7**	300, 360, 650	78	2**L**^**3**^+3Cl_2_+6H_2_O	22	PdO + Few carbons

**8**	380, 620, 800	82	2**L**^**4**^+2Cl_2_+4H_2_O	18	PdO + Few carbons

**Table 4:** Inhibition zone diameter of free ligands and its Pt(IV) and Pd(II) complexes.

Sample		Inhibition zone diameter (mm/mg Sample)			
		Bacteria		Fungi	
		*Escherichia coli* (G^−^)	*Staphylococcus aureus* (G^+^)	*Aspergillus flavus* (Fungus)	*Candida albicans* (Fungus)
Standard	Ampicillin: Antibacterial agent	30	24	--	--
	Amphotericin B: Antifungal agent	--	--	16	21

Control: DMSO		0.0	0.0	0.0	0.0

**L** ^**1**^		11	10	10	0.0

**L** ^**2**^		0.0	0.0	0.0	0.0

**L** ^**3**^		0.0	0.0	0.0	0.0

**L** ^**4**^		15	10	0.0	0.0

**1**		19	18	16	0.0

**2**		12	11	0.0	0.0

**3**		24	23	12	0.0

**4**		14	12	0.0	0.0

**5**		14	15	15	0.0

**6**		11	11	0.0	0.0

**7**		16	17	12	0.0

**8**		14	15	11	0.0

*∗*Ampicillin and amphotericin B are standards of antibacterial and antifungal agents.

**Table 5:** IC_50_ activity of the free ligands and its Pt(IV) complexes.

Against human colon cancer cell lines								

Concentration (*μ*g/mL)	Viability (%)							
	**L** ^**1**^	**1**	**L** ^**2**^	**2**	**L** ^**3**^	**3**	**L** ^**4**^	**4**

100	65	66	61	72.7	55.3	6.4	35.5	44.6

50	70	67.8	64.4	81.7	60	31.8	41.4	56.6

10	72	70	69.3	82.5	65	62.9	66.8	71.5

IC_50_	170	461	277	259	162.8	27	50.7	79

Against human lung cancer A549 cell lines								

Concentration (*μ*g/mL)	Viability (%)							
	**L** ^**1**^	**1**	**L** ^**2**^	**2**	**L** ^**3**^	**3**	**L** ^**4**^	**4**

150	82.4	100	96	100	100	44.6	100	77.7

100	83.4	100	98.7	100	100	61.7	100	100

50	86	100	100	100	100	66.5	100	100

10	98.7	100	100	100	100	100	100	100

IC_50_	431.4	-	1305.7	-	-	128	-	212

## Data Availability

The data used to support the findings of this study are available from the corresponding author upon request.
